# Multiphotonic Tuning of Nonlinearities Exhibited by Plasma Polypyrrole

**DOI:** 10.3390/jfb17070341

**Published:** 2026-07-14

**Authors:** Carlos Alberto Espinoza-Garcés, Victor Manuel Garcia-de-los-Rios, Axayacatl Morales-Guadarrama, Christopher René Torres-SanMiguel, Carlos Torres-Torres

**Affiliations:** 1 Sección de Estudios de Posgrado e Investigación, Escuela Superior de Ingeniería Mecánica y Eléctrica Unidad Zacatenco, Instituto Politécnico Nacional, Mexico City 07738, Mexico; 2 Departamento de Ingeniería Eléctrica, Universidad Autónoma Metropolitana Unidad Iztapalapa, Mexico City 09340, Mexico

**Keywords:** nonlinear optics, Z-scan, Kerr effect, nonlinear optical absorption, nonlinear refractive index, Polypyrrole

## Abstract

Polypyrrole (PPy) synthesized via plasma polymerization (PPPy) offers a unique combination of electrical conductivity, biocompatibility and stability. This advanced material has emerged as a promising platform for next-generation optoelectronics and multiphotonic biosensors. However, fully unlocking its potential has been hindered by processing challenges that restrict the fabrication of tailored specimens for precise optical and mechanical characterization. This work overcomes these limitations by isolating and analyzing the nonlinear optical (NLO) response of PPPy across three distinct architectural paradigms: Electrospinning, Coating on SiO_2_ Slides, and Dust of Polymer. Using open- and closed-aperture Z-scan techniques, we demonstrate that PPPy exhibits highly pronounced, architecture-dependent NLO behaviors. Notably, the Electrospinning PPPy morphology triggered a full order-of-magnitude enhancement in the nonlinear refractive index (n_2_) alongside low-threshold nonlinear absorption (β × 10^−8^ cm/W). Irradiance-dependent properties further revealed an optical anisotropy, directly governed by the structural and morphological orientation inherent to each processing method. Since optical nonlinearities are closely related to mechanical and electronic properties, these findings provide a critical blueprint for developing macromolecular architectures, opening new pathways for biocompatible cutting-edge multiphotonic platforms, innovative coatings and surface modifications for tailored implants.

## 1. Introduction

Polypyrrole (PPy) is one of the most studied conjugated polymers due to its excellent electrical conductivity, biocompatibility, and stability under ambient and thermal conditions, as well as its simple synthesis process [1]. There are several polymerization methods; the most prominent correspond to chemical polymerization, Electrospinning, ultrasonication, electrochemical polymerization, and photopolymerization. For the preparation of PPy, chemical and electrochemical polymerization are primarily used [2]. Recently, plasma polymerization has been employed to obtain PPy on particles or thin films [3]. The main applications of these materials include the development of biosensors [4], the use of nanofibers in engineering projects [5], and the use of scaffolding elements in the treatment of traumatic spinal cord injury [6]. The mechanical properties of PPy are directly related to the type of synthesis used for its generation. Some researchers have reported results that involve an empirical method to evaluate these properties, such as elastic moduli of approximately 100 kPa [7], or 360 MPa when it is used with supporting elements [8]. Characterizing the mechanical behavior makes it suitable for use in computational models and numerical simulations, which aim to theorize the results of the material’s various applications.

However, to date, specific values of physical properties for pure PPy synthesized via plasma polymerization (PPPy) have not been reported. Some experimental and analytical methodologies have been employed to estimate values for physical parameters. Among these, the use of acoustic, nanometric, and optical techniques is highlighted. Acoustic methods quantify an approximation for dynamic elastic moduli; an example of this is the use of cell phones and applications to determine the dynamic elastic moduli of carbon steel and aluminum plates [9]. Another method is nanoindentation, which consists of applying controlled forces to a material in order to evaluate its mechanical properties at the nanoscale [10]. Finally, optical techniques are used to characterize the density and nonlinear optical properties of materials, such as interferometry or Z-scan measurements [11]. In linear optics, when a beam of light of low or moderate intensity irradiates a material, the wave properties, such as wavelength or frequency, are not affected by its interaction with the material. Reflection, absorption, and refraction are some examples of linear optics phenomena [12]. Nonlinear optics, on the other hand, encompasses a variety of nonlinear responses that occur when high-intensity light propagates through a medium, causing irradiance-dependent effects. The most representative example is the Kerr effect, which describes the refractive index of materials as a function of light intensity [13].

The Z-scan method is particularly useful for measuring the magnitude and sign of the third-order optical nonlinearities of dielectric materials, including crystals, organic materials and semiconductors [14]. The optical properties of materials arise from high-intensity irradiation, and they are a direct consequence of the interaction between intense electromagnetic fields and the material [15]. Particularly, a Z-scan analysis serves as an efficient tool for the identification and characterization of the nonlinear optical absorption coefficient and nonlinear refractive index [16]. One of the main advantages of quantifying optical nonlinearities using the Z-scan technique is the reduction in the use of multiple lasers, which is mandatory when elliptical polarization, multi-wave mixing, or the third harmonic method is employed. Typically, the nonlinear refractive index tends to decrease with a lower concentration of the component [17]. An important limitation lies in the sample type, affecting the sensitivity of nonlinear optical characterization. However, PPy has been reported to exhibit nonlinear absorption, nonlinear scattering effects, and birefringence [18]. On the other hand, systematic approaches have been proposed to evaluate the uncertainty in Z-scan measurements, where parameters such as optical power and beam size represent the main sources of error. However, these approaches are based on ideal Gaussian models and do not consider structured beams or non-local effects, which limit their applicability [19]. The nonlinear properties of PPy have been reported when synthesized by chemical oxidation. However, this characterization has been carried out in the presence of ionic agents or copolymers [20], with the Z-scan technique yielding a value of 0.11 × 10^−7^ cm^2^/W for the nonlinear refractive index. Meanwhile, a value of 0.14 for the absorption coefficient has been reported [21]. Characterization of mechanical properties by optical methods could be considered non-destructive experiments on the material, along with mathematical models that accurately quantify their properties. An example of such a mathematical model is the one described for estimating yield strength in relation to refractive index [22]. On the other hand, some research evaluates the influence of surface morphology changes [23] or the synergistic effects on titanium alloy under tribocorrosion conditions [24]. Another one evaluates the nonlinear response of NiSe_2_ by optimizing cavity dispersion [25]. Another method used for the optical characterization of a material has been employed through the development of SPASER, the result of which describes the amplification of plasmons [26]. Finally, the analytical double-hump solutions demonstrate their potential to enhance soliton intensity in dispersion-decreasing optical fiber systems [27].

Previous reports focused on the characterization of the mechanical properties exhibited by PPPy provide further validation for the relationship between a support material and its coating. However, the development of tensile tests is limited by the production and feasibility of manufacturing pure polymer specimens. In addition, the direct synthesis of the polymer in the specimen raises the possibility of plastic deformation due to current induction. In this direction, this research has been devoted to characterizing nonlinear optical properties by implementing a Z-scan technique on three different types of samples of PPPy. Our results indicate a remarkable difference in the third-order optical nonlinearities related to each sample and associated with isotropic or anisotropic behavior in PPPy, which was assumed to be derived from the synthesis route for their fabrication.

## 2. Materials and Methods

For each computational tool as Comsol Multiphysics (Burlington, MA, USA) that develops finite element analysis, it is essential to specify key material parameters such as elastic modulus, Poisson’s ratio, or tangential moduli for mechanical properties. Electrical and optical properties can be associated when electromagnetic field simulation is considered; generally, the values of these variables are experimentally obtained. In general terms, the methodology in this research is classified into three stages: first, sample selection and optical evaluation; second, analytical evaluation; and finally, experimental Z-scan analysis.

### 2.1. PPPy Samples, Synthesis and UV-Vis

**Figure 1:**
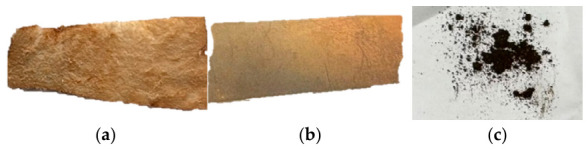
Polymeric samples: (**a**) PPPy Electrospinning, (**b**) PPPy Coating on SiO_2_ Slide, and (**c**) PPPy Dust.

PPPy was synthesized using plasma oxidation, which was performed in a cylindrical Pyrex reactor. This reactor consists of an electrical circuit with two electrodes where polymer particles are deposited or which, alternatively, serve as circuit terminals. A sample support material (polylactic acid, PLA, or a glass slide) is connected to the reactor, and its surface serves as the polymerization base. Plasma oxidation requires two main reagents: pyrrole monomer with a purity of 96% and iodine as a doping agent. Both reagents are fed in an equimolar manner at a pressure of 72 Pa, and 30 kV is induced in the circuit for 30 min [3]. For the purposes of this research, three kinds of samples were used to evaluate their nonlinear refractive index and nonlinear optical absorption coefficient. For each sample, a thickness of around 50 µm was considered [28]. In addition, thickness measurements were developed with structural characterization on an Olympus GX 51 electronic microscope (Hachioji, Tokyo, Japan). The optical transmittance spectrum of each sample was obtained using a PerkinElmer XLS UV spectrophotometer (Waltham, MA, USA). Figure 4 describes the optical absorbance for each sample. Three different PPPy samples were prepared and considered for evaluation by a Z-scan technique: PPPy Electrospinning, PPPy Coating on a SiO_2_ Slide and PPPy Dust. The particle size, final implant process and coated slide can be visually observed in Figure 1.

- PPPy Electrospinning: As a first step, the Electrospinning implant is developed, taking as a base polylactic acid (PLA). Subsequently, the Electrospinning sample is induced in the plasma reactor, with the deposition of PPPy, synthesized under the operating conditions for 1 h at 20 kV. The Electrospinning fibers allow the study of the influence of anisotropic and oriented structures on the nonlinear optical response of the polymer; for a graphical representation, see Figure 1a.
- PPPy Coating on SiO_2_ Slide: Under this method, the SiO_2_ slide is used as the polymerization surface. One end is connected as the positive pole, and the opposite end as the negative pole. Polymerization is not uniformly distributed due to the conduction of current within the slide. For 45 min, 35 kV is applied to the reactor. The polymer on the slide has a yellowish appearance, with subtle variations and small gaps throughout the sample. The flat and homogeneous geometry promotes beam propagation and reduces multiple scattering; an example of this is represented in Figure 1b.
- PPPy Dust: In this stage, PPPy particles are adhered to the electrode walls, forming a continuous polymer layer, at original conditions (30 min and 30 kV). To remove the PPPy from the electrodes, it is necessary to scrape the electrode surface with a small spatula, taking care in order to avoid damage. The resulting product is a dark grayish material of 15 mg, which is collected in a sampling bottle. The sample for Z-scan analysis is prepared with a fragment of the product and is placed on a slide, with drops of distilled water added for uniform distribution. Finally, it is covered with a coverslip; this type of sample allows for the analysis of the average response of the material in particulate form. This is represented in Figure 1c.

### 2.2. Nonlinear Optical Characterization by Z-Scan Analysis

The open- and closed-aperture Z-scan method can be used to characterize the nonlinear refractive index and nonlinear optical absorption coefficient [29]. The nonlinear absorption coefficient (β), which is related to the irradiance and the effective length of the sample, can be quantified from Equation (1).

(1) $$T_{0}\left(Z_{0},\Delta \phi_{0}\right)=1-\frac{\beta I_{0}L_{eff}}{2\sqrt{2(1+\left(x^{2}\right))}}$$

where T_0_ is the normalized transmittance, β is the nonlinear absorption coefficient, $I_{0}\;$is the normalized irradiance, $L_{eff}$ is the effective length of the sample, and $x^{2}$ is the ratio of the sample displacement with respect to the focal point [Z2Z02].

The nonlinear refractive index of a material is associated with its nonlinear phase $\Delta \phi_{0}$ and can be represented by Equation (2).

(2) $$\Delta \phi_{0}=k\Delta n_{0}L_{eff}$$

where $\Delta \phi_{0}$ is the nonlinear phase, k=2πλ, $\Delta n_{0}$ relates the product of the maximum irradiance at the focal point and the nonlinear refractive index ($n_{2}$), and L_eff_ is the effective length of the sample. However, the optical phase shift is also associated with the estimation of the normalized transmittance at closed aperture, which can be estimated using Equation (3).

(3) $$T_{0}=1-\frac{4\Delta \phi_{0}(x)}{\left(x^{2}+9)(x^{2}+1\right)}$$

where $T_{0}$ is the normalized transmittance value, $\Delta \phi_{0}$ is the optical phase change and *x* is the ratio of the sample displacement with respect to the focal point (ZZ0), Z0=kw022; this value is 0.8503 mm, and $w_{0}$ is the beam radius of 0.014 mm.

In order to analyze the accuracy of the nonlinear properties, an evaluation was performed using a programming code developed in MATLAB R2023a (Natick, MA, USA) software. The main parameter used was displacement within a range of −20 to 20 mm, with the focal point as the origin, distributing this range across 1000 data points. Before developing each measure, the Z-scan experimental setup was calibrated using a carbon disulfide (CS_2_) sample, where a direct correlation was made with the consulted literature that reports a nonlinear refractive index value of 3.2 × 10^−19^ m^2^/W [30]. The relative error with respect to our calibration was 12.1%. Subsequently, the normalized transmittance behavior was quantified again in closed and open apertures, respectively. Finally, to estimate the value of the nonlinear refractive index, it is enough to express Equation (4) as,

(4) $$n=n_{0}+n_{2}I$$

where n is the value of the refractive index, $n_{0}$ is the value of the linear refractive index, $n_{2}$ is the value of the nonlinear refractive index and I is the irradiance ratio.

The linear refractive index was calculated by Herve’s and Vandamme’s mathematical model, as Equation (5) [31],

(5) $$n_{0}=\sqrt{1+{\left(\frac{A}{E_{g}+B}\right)}^{2}}$$

where n_0_ represents the linear refractive index on the sample; the system constants in our case correspond to *A* = 13.6 (eV), which denotes the hydrogen ionization energy; *B* = 3.4 (eV) represents the difference between the UV resonance energy; and *E_g_* rep-resents the bandgap evaluated experimentally with the absorption coefficient.

**Figure 2:**
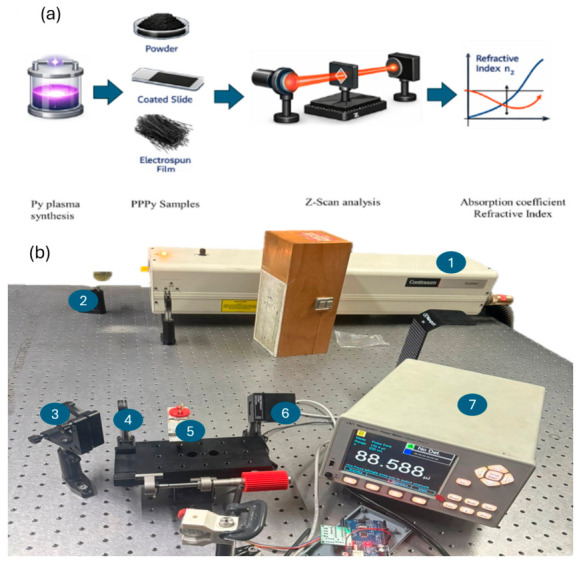
Z-scan experimental setup: (**a**) scheme; (**b**) photo.

A scheme and a photo of our Z-scan experiment are shown in Figure 2.

In order to conduct the Z-scan analysis, an optical laser source with a Gaussian beam profile is required. In this investigation, we used a Nd:YAG high-powered laser (Continuum SL II-10, Cambridge, MA, USA) at 532 nm. A biconvex focusing lens focuses the laser beam at a specific point (focal spot) where the intensity is maximum. The energy laser pulse width was 4 ns, the focal length of the lens was 150 mm, the laser focus 1/e-fluence diameter was 0.014 mm and the Rayleigh length was 0.8503 mm. A displacement platform provides the space to hold the PPPy sample, and it is able to move across the focal spot. Also, a diaphragm (aperture) can be placed in the far field, behind the lens and sample. This diaphragm measures the transmittance as the sample moves across the focal spot, commonly referred to as the aperture being closed and opened, the refractive index and the nonlinear absorption coefficient. Finally, a photodetector is connected to a computer that records the data, such as displacement, intensity, and transmittance. A scheme is illustrated in Figure 2b, where the following numbers identify each component in the Z-scan arrangement: (1) pulsed-type laser, (2,3) reflective mirrors, (4) focal lens, (5) PPPy sample, (6) diaphragm and (7) oscilloscope. Prior to performing the analysis on each PPPy sample, the ablation process was experimentally measured on PPPy. Finally, closed-aperture Z-scan and open-aperture Z-scan experiments were undertaken below this energy level. The procedure involves placing the sample at the focal point and directing the laser beam, gradually increasing its intensity until the slightest interaction with the material is detected. Some variations on each measured variable, such as energy (4%), laser radius (4%), transmittance (3%) and effective length (2%), are considered to develop an uncertainty analysis (M^2^) according to mathematical expression no. 6.

(6) $$M^{2}=\frac{u{\left(E\right)}^{2}}{E}+\frac{u{\left(T\right)}^{2}}{T}+\frac{u{\left(w_{0}\right)}^{2}}{w_{0}}+\frac{u{\left(L\right)}^{2}}{L}$$

## 3. Results

**Figure 3:**
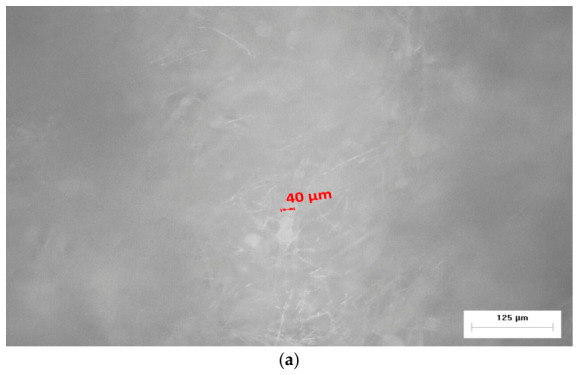

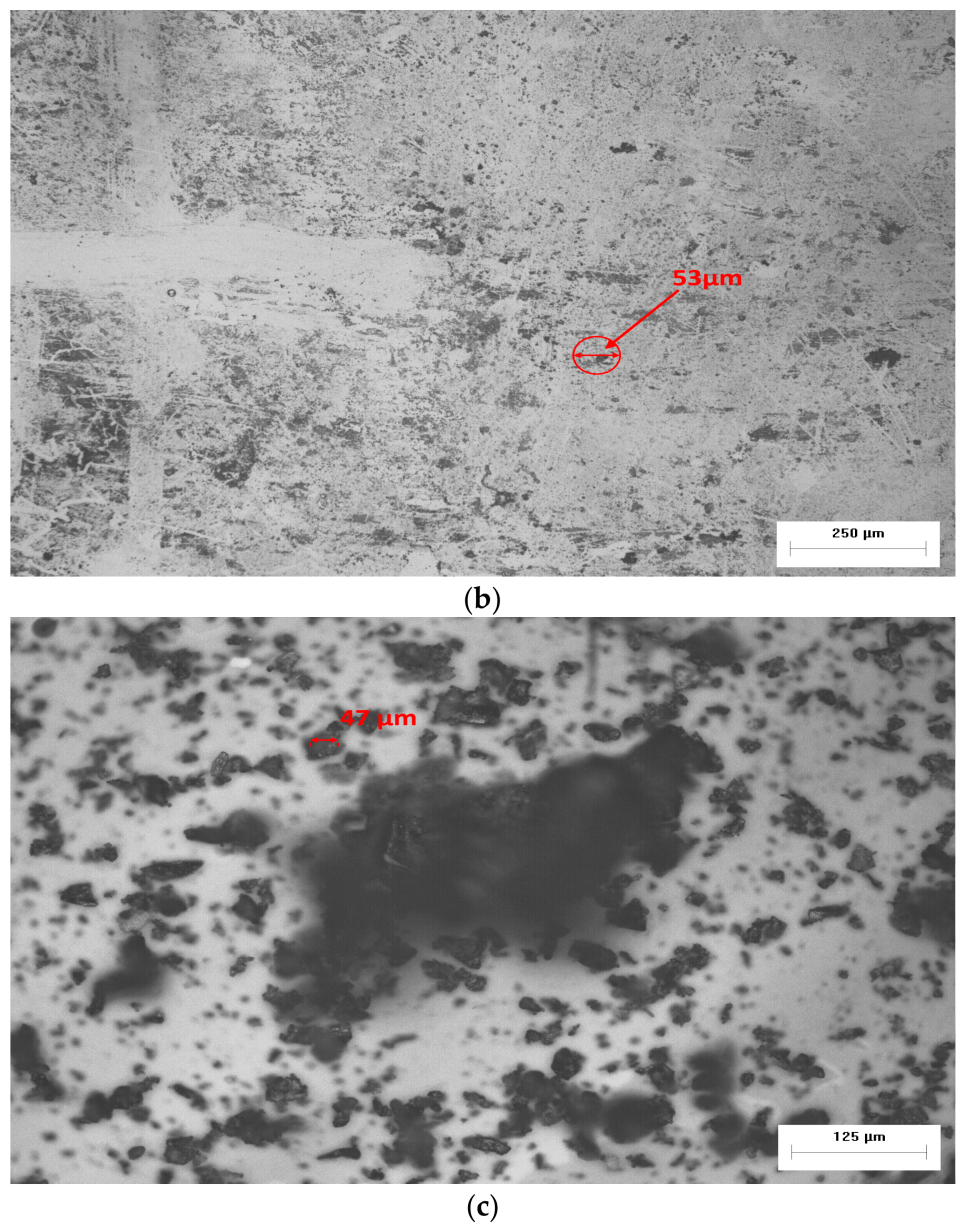
Micrographs of samples: (**a**) PPPy Electrospinning, (**b**) PPPy Coating on SiO_2_ Slide and (**c**) PPPy Dust.

PPy synthesized by plasma oxidation yields higher-quality samples, avoiding impurities from solutions that are easily present in electro-polymerization or chemical oxidation. Our first result describes the size of each kind of sample. The micrographs illustrated in Figure 3 show differences in geometry and quality among PPPy samples. The fiber radius in PLA for PPPy Electrospinning is shown in Figure 3a (40 µm), the thickness of PPPy Coating on SiO_2_ Slide is shown in Figure 3b (53 µm), and the crystal size of PPPy Dust can be seen in Figure 3c (47 µm).

**Figure 4:**
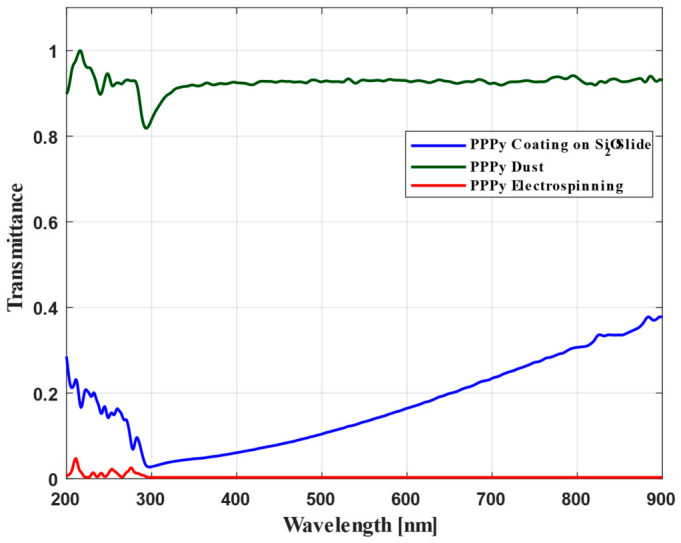
UV-Vis results for PPPy samples.

Spectroscopic analysis shows stronger absorption below 350 nm; however, some saturation effects may be present in the Electrospinning sample due to the presence of the base material, PLA. On the other hand, in samples such as the PPPy Dust deposition, absorption is low. Finally, the sample coated directly with PPPy shows a clean transmittance spectrum, free of materials that alter or interfere with it. Figure 4 graphically plots the spectra of the PPPy samples.

**Figure 5:**
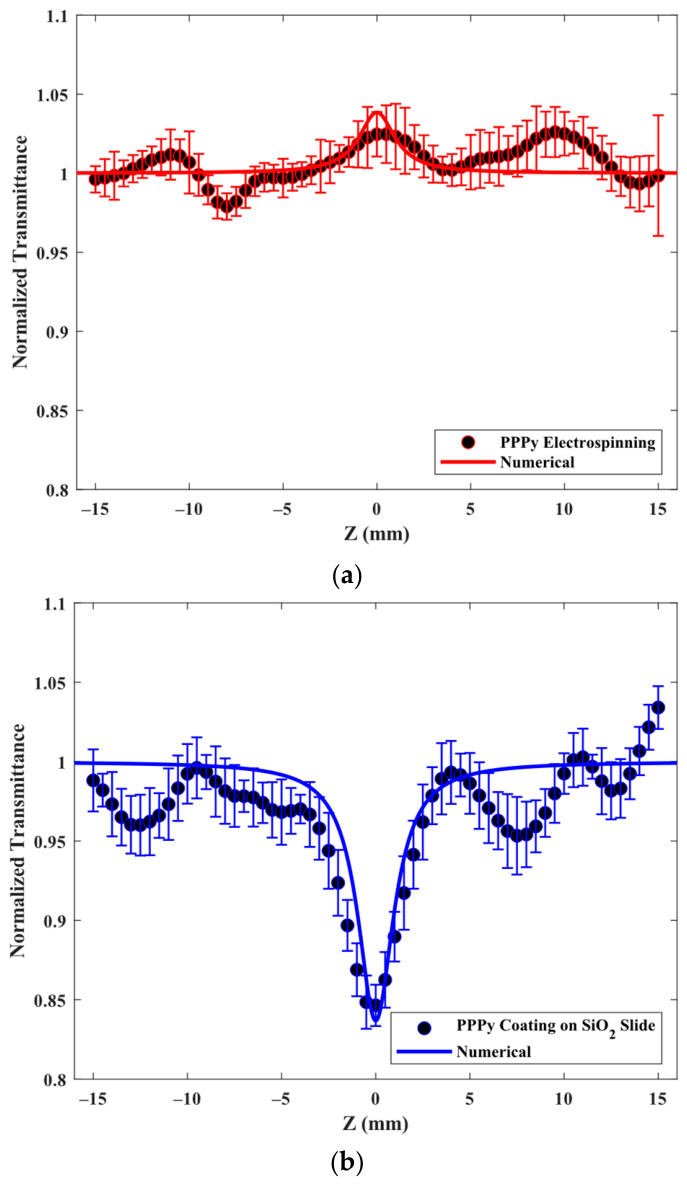

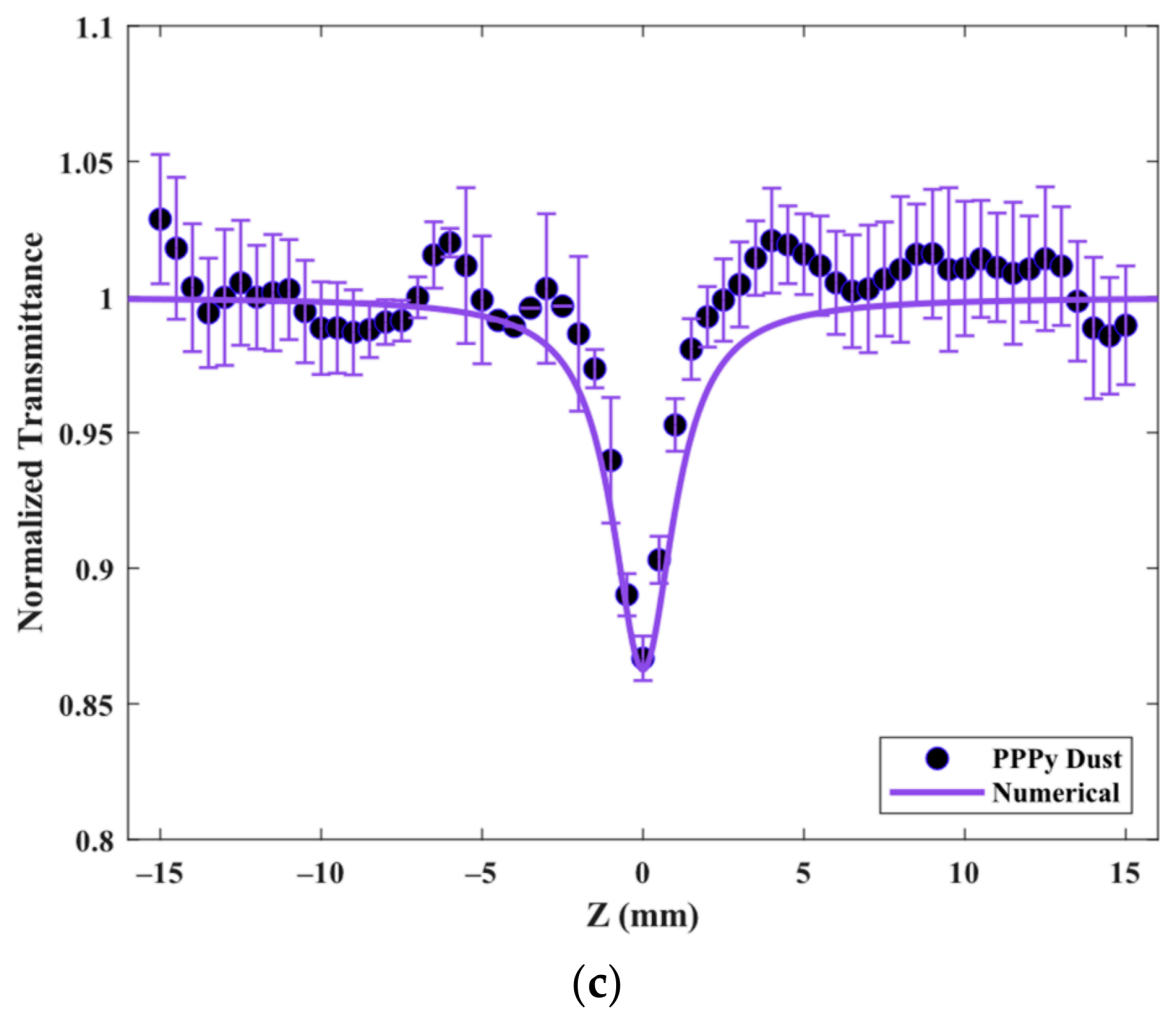
Nonlinear transmittance of samples for open-aperture Z-scan traces: (**a**) PPPy Electrospinning (20 μJ), (**b**) PPPy Coating on SiO_2_ Slide (107 μJ) and (**c**) PPPy Dust (60 μJ).

In the open-aperture Z-scan, the peak value of higher intensity is the focal point. The displacement behavior around the focal point is described in Figure 5 for the PPPy Electrospinning (Figure 5a), PPPy Coating on SiO_2_ Slide (Figure 5b) and PPPy Dust (Figure 5c) samples.

**Figure 6:**
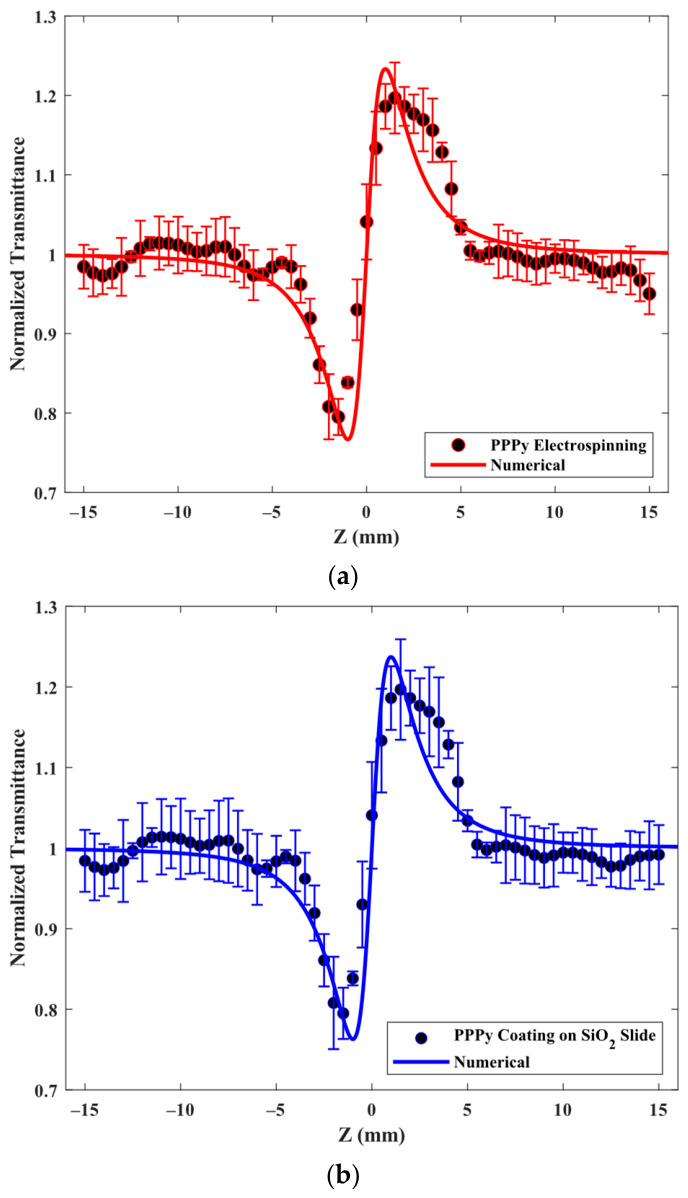

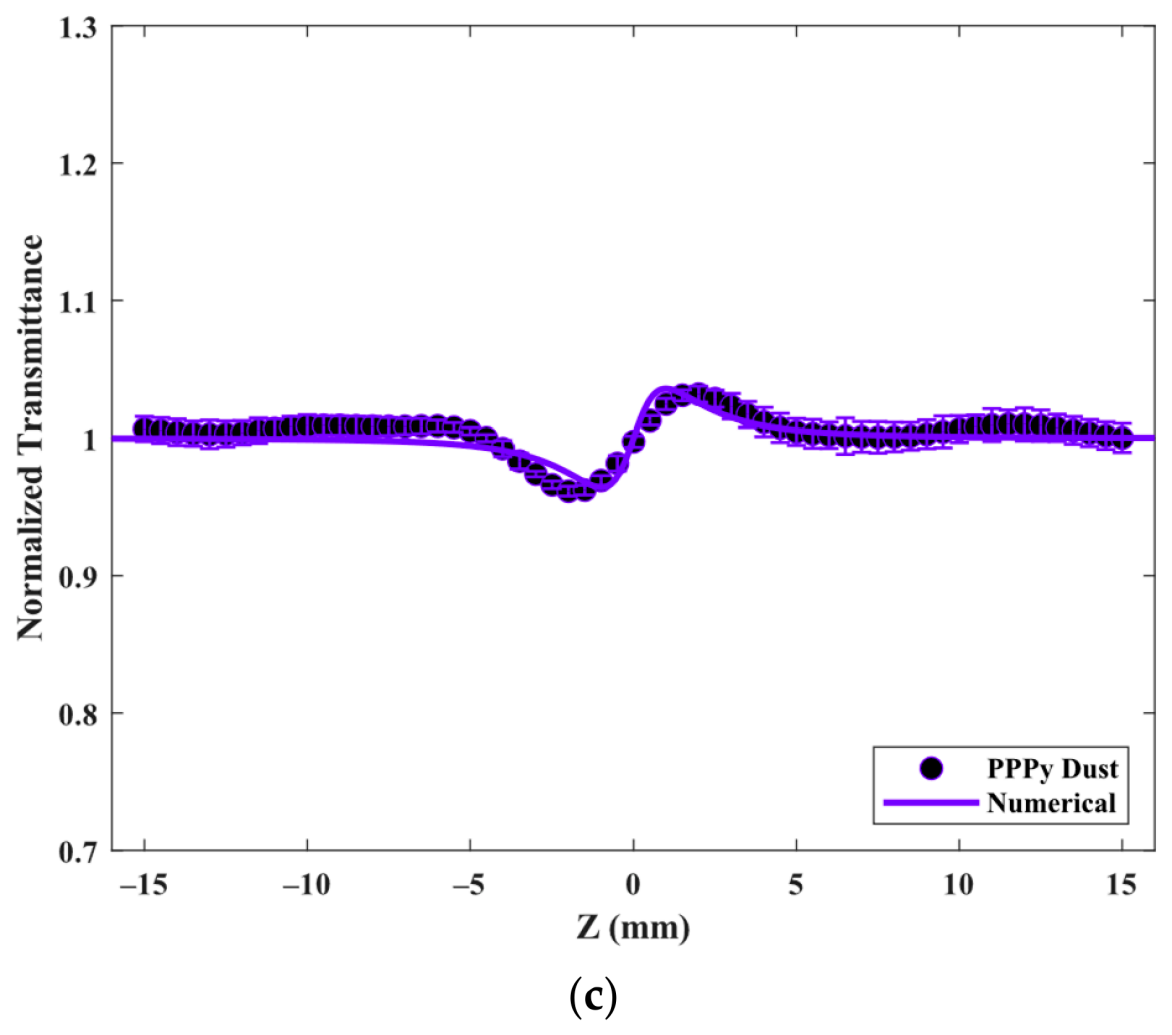
Nonlinear transmittance of samples for closed-aperture Z-scan traces: (**a**) PPPy Electrospinning (22 μJ), (**b**) PPPy Coating on SiO_2_ Slide (22 μJ) and (**c**) PPPy Dust (37 μJ).

Similarly, the closed-aperture Z-scan technique results are described in Figure 6 for PPPy Electrospinning (Figure 6a), PPPy Coating on SiO_2_ Slide (Figure 6b) and PPPy Dust (Figure 6c).

Table 1 presents the nonlinear optical parameters. The significant variations observed in these magnitudes (n2, β and ablation threshold), among the different PPPy morphologies, suggest an anisotropic optical response. This is associated with structural and morphological differences induced by the fabrication method; on the other hand, Table 2 shows the uncertainty analysis for each sample.

## 4. Discussion

The versatility of PPPy, both in Electrospinning and particle lattices, determines its macroscale NLO responses, offering an adjustable platform for advanced photonic applications. Elucidating these properties requires critical contextualization within the landscape of contemporary NLO materials. In the specific case of PPy samples, their nonlinear response does not depend on morphology; the material’s substrate and thermal effects may also play a role. The value of linear properties decreases in the polymer powder sample, and this decrease is attributed to various variables such as particle size and position. This phenomenon is heavily governed by the local irradiance thresholds and structural confinement. While the particulate Dust was evaluated at a lower energy threshold (37 µJ), the Electrospinning sample sustained a higher intensity (22 μJ), driving the highly oriented macromolecular chains and efficient thermal dissipation of fibrous geometries. This intensity-dependent scaling mirrors observations in conventional nanocomposites, such as polymethyl methacrylate zinc oxide (PMMA-ZnO) systems, where NLO parameters scale proportionally with continuous light intensity due to localized field [32,33]. However, unlike conventional polymerization, which suffers from nanoparticle agglomeration at higher weight fractions (1 to 15 wt%), our pristine PPPy architectures exhibit intrinsic, matrix-free optical tuning governed solely by morphological manipulation. Furthermore, the structural integrity and stability of PPPy under variable laser irradiation highlight its superiority over carbon-based nanomaterials. In graphene oxide suspensions synthesized via the modified Hummers method, the refractive index and nonlinear absorption coefficients typically degrade as beam incidence increases, a drawback exacerbated by chemical impurities from residual synthesis reagents [34]. Conversely, our PPPy samples maintained a stable and reproducible nonlinear refractive index across varying light intensities. This optical robustness is a direct consequence of the highly cross-linked, pinhole-free network inherent to plasma polymerization, which eliminates the need for chemical solvents or catalysts, minimizing the optical scattering and photodegradation pathways.

In contrast, our systematically varied irradiance profiling explicitly maps the dynamic third-order nonlinear evolution of PPPy, mapping its anisotropic nature across specific physical coordinate states. Given this exceptional architecture-tailored NLO response combined with intrinsic biocompatibility, PPPy bridges the gap between material synthesis and cutting-edge biophotonics. The high optical-damage threshold and profound third-order sensitivity demonstrate that these platforms can be seamlessly integrated into next-generation all-optical biosensors [35], precise noninvasive multiphotonic imaging, and light-triggered biomedical devices [36], establishing a new paradigm for biocompatible light-controlled technologies [37].

## 5. Conclusions

The advantages offered by plasma oxidation compared to other preparation methods lie particularly in not using other agents that contaminate or do not favor polymerization involved in coating technology. Unfortunately, the amount of energy is large, and the product obtained is not usually sufficient to develop significant test specimens, if common tensile tests are considered. In this work, it is indicated by the UV-Vis spectrum that higher absorption can be obtained in PPPy due to the presence of the base component (PLA). In contrast, the spectrum of the powdered sample shows low absorption, possibly due to the absence of the polymer, since distilled water was used as the medium for sample adhesion to avoid contact with the solvent (ketone). The order of the absorption coefficient on the PPPy Electrospinning sample shows a possible saturable absorption effect; this discrepancy is possibly associated with the presence of PLA elements. The excitation of the polymer particles themselves could have a direct correlation with the photosensitivity of the compound. There is a possibility that when exposed to the laser beam, the PPPy particle or segment maintains its energetic excitation for a period. However, although each sample was evaluated at similar light intensities, the purpose is to provide feedback, assess nonlinear behavior under similar conditions, and broaden the analysis range. The refractive index values for each sample describe a similar behavior. As a result, it is possible to theorize the potential orthotropic behavior of the material, with potential applications developing high-tech coatings and implants assisted by dynamic surface modifications dependent on the energy deposited.

## Figures and Tables

**Table 1:** Nonlinear results for absorption coefficient and refractive index.

PPPy Samples	Ablation [J/cm^2^]	I_0_ Closed-Zscan [W/cm^2^]	β [cm/W]	I_0_ Open-Zscan [W/cm^2^]	n_2_ [cm^2^/W]	*n*
Electrospinning	0.430	8.12 × 10^8^	9.55 × 10^−7^	8.93 × 10^8^	−2.25 × 10^−11^	1.48
Coating on SiO_2_ Slides	0.392	4.34 × 10^9^	1.22 × 10^−7^	8.93 × 10^8^	−2.25 × 10^−11^	1.47
Dust	0.248	2.44 × 10^9^	1.84 × 10^−7^	1.50 × 10^9^	−1.13 × 10^−12^	1.49

**Table 2:** Uncertainty analysis for PPPy samples.

PPPy Samples	M^2^ (β) [cm/W]	M^2^ (n^2^) [cm^2^/W]
Electrospinning	9.55 × 10^−7^ ± 0.06	−2.25 × 10^−11^ ± 0.08
Coating on SiO_2_ Slides	1.22 × 10^−7^ ± 0.06	−2.25 × 10^−11^ ± 0.08
Dust	1.84 × 10^−7^ ± 0.06	−1.13 × 10^−12^ ± 0.08

## Data Availability

The original contributions presented in this study are included in the article. Further inquiries can be directed to the corresponding authors.
